# The Fitter the Better? Cardiopulmonary Exercise Testing Can Predict Pulmonary Exacerbations in Cystic Fibrosis

**DOI:** 10.3390/children8060527

**Published:** 2021-06-21

**Authors:** Asterios Kampouras, Elpis Hatziagorou, Thomas Kalantzis, Vasiliki Avramidou, Kalliopi Kontouli, Fotios Kirvassilis, John Tsanakas

**Affiliations:** 1 Pediatric Pulmonology and CF Unit, 3rd Department of Paediatrics, Hippokration Hospital, Aristotle University of Thessaloniki, 541 24 Thessaloniki, Greece; elpcon@otenet.gr (E.H.); vavramid@yahoo.gr (V.A.); kkontoul@otenet.gr (K.K.); fkirvas@otenet.gr (F.K.); tsanakas@hol.gr (J.T.); 2 Hellenic Statistics Authority, 185 10 Piraeus, Greece; tkalant@uom.edu.gr

**Keywords:** cystic fibrosis, exercise testing, pulmonary exacerbation, prognosis

## Abstract

Background: The role of cardiopulmonary exercise testing (CPET) in the assessment of prognosis in CF (cystic fibrosis) is crucial. However, as the overall survival of the disease becomes better, the need for examinations that can predict pulmonary exacerbations (PEx) and subsequent deterioration becomes evident. Methods: Data from a 10-year follow up with CPET and spirometry of CF patients were used to evaluate whether CPET-derived parameters can be used as prognostic indexes for pulmonary exacerbations in patients with CF. Pulmonary exacerbations were recorded. We used a survival analysis through Cox Regression to assess the prognostic role of CPET parameters for PeX. CPET parameters and other variables such as sputum culture, age, and spirometry measurements were tested via multivariate cox models. Results: During a 10-year period (2009–2019), 78 CF patients underwent CPET. Cox regression analysis revealed that VO_2_peak% (peak Oxygen Uptake predicted %) predicted (hazard ratio (HR), 0.988 (0.975, 1.000) *p* = 0.042) and PetCO_2_ (end-tidal CO_2_ at peak exercise) (HR 0.948 (0.913, 0.984) *p* = 0.005), while VE/VO_2_ and (respiratory equivalent for oxygen at peak exercise) (HR 1.032 (1.003, 1.062) *p* = 0.033) were significant predictors of pulmonary exacerbations in the short term after the CPET. Additionally, patients with VO_2_peak% predicted <60% had 4.5-times higher relative risk of having a PEx than those with higher exercise capacity. Conclusions: CPET can provide valuable information regarding upcoming pulmonary exacerbation in CF. Patients with VO_2_peak <60% are at great risk of subsequent deterioration. Regular follow up of CF patients with exercise testing can highlight their clinical image and direct therapeutic interventions.

## 1. Introduction

Cardiopulmonary exercise testing (CPET) provides a thorough assessment of pulmonary, cardiovascular, and muscular systems, helping to distinguish the system most responsible for exercise intolerance. In respiratory disorders, CPET can help recognize the pathophysiology that can lead to exercise intolerance, assess therapeutic interventions, and provide key data on prognosis [1]. 

In cystic fibrosis (CF), the prognostic value of CPET was firstly described in 1992 by the landmark paper of Nixon et al. [2], where VO_2_peak was found to be a significant predictor of mortality in CF. Since then, various studies have confirmed the prognostic role of VO_2_peak, respiratory equivalent for oxygen at peak exercise (VE/VO_2_), and other parameters [3,4,5,6] in CF. In accordance with others with these findings, CPET is advised to be part of the annual routine CF evaluation [7]. 

Hence, overall survival in CF has increased dramatically since 1992 [8], and interest has shifted toward recognizing potential predictors of pulmonary exacerbations in order to prevent worsening in patients’ clinical condition [9].

A pulmonary exacerbation (PEx) in CF is preceded by various physiological alterations that, in many cases, are clinically obvious even more than one month before the worsening of symptoms [10]. As clinical symptoms can become apparent so early, it is reasonable that pathophysiological remodeling along with a higher possibility for a mild gas exchange impairment [11] could be present even earlier. On that basis, possible alterations in CPET indexes such as VO_2_peak, VE/VO_2_, VE/VCO_2_, and others that serve as markers of elevated effort to absorb oxygen could hint at underlying mechanisms responsible for an upcoming PEx.

Therefore, our study’s primary aim was to examine the relationship between CPET-derived parameters and the future occurrence of pulmonary exacerbations in CF.

## 2. Methods

### 2.1. Study Design and Subjects

This was a mixed retrospective and prospective study. A total of 78 patients (aged >9 years old) followed by our CF Unit performed full cardiopulmonary tests one to three times per year, during a 10-year period (2009–2019). Patients that were started on CFTR modulator were not included in the analysis, as this could have biased our results. A pulmonary CF exacerbation was defined as the need for additional treatment when the following occurred: (i) alteration in color or quantity of sputum, (ii) increase in cough, (iii) anorexia or malaise, (iv) decrease in pulmonary function by more than >10% (v) shortness of breath, or (vi) alteration in radiographic findings [12]. In total, 265 cardiopulmonary exercise tests were performed in a 10-year period. Of these, 248 fulfilled the criteria of a maximal test. The study was approved by the respective ethics committee, and written informed consent was obtained from all patients (IRB File No.:3/2-5-2018).

### 2.2. Spirometry

Spirometry was performed with a Vitalograph spirometer (Vitalograph 2120 electronic spirometer, Vitalograph Ltd. Ennis, Ireland) according to established standards [13]. All values were measured and expressed in % predicted using the Global Lung-Function Initiative (GLI 2012, http://www.lungfunction.org, date accessed on 12 June 2021).

### 2.3. Cardiopulmonary Exercise Testing

CPET was performed on a cycle ergometer (Ergoline, Vmax Series V20-1, SensorMedics, Hünenberg, Switzerland) with simultaneous electrocardiography and blood pressure monitoring (cardiograph model: Corina, S. No.: 101164361, Cardiosoft software V5.15, GE Medical Systems Information Technologies GmbH, Freiburg, Germany). Godfrey protocol [14] was applied for the exercise testing: 2 min of resting measurements were followed by 2 min of cycling on 10 Watts, and afterward—for the exercise phase—workloads were increased according to the patient’s height. For patients <120 cm, workload was 10 W/min; for 120–150 cm tall, 15 W/min; and patients >150 cm, workload was increased by 20 W/min until volitional fatigue, with test duration between 8 and 12 min. A test was considered maximal if heart rate (HR) > 85% predicted (14) and respiratory exchange ratio (RER) >1.05 [15]. The following parameters were calculated: VO_2_peak, ventilatory equivalents for oxygen and carbon dioxide at peak exercise (VE/VO_2_ and VE/VCO_2_ respectively), and breathing reserve (BR). VO_2_peak % predicted was calculated using Orenstein’s method:

Girls: V′O_2_peak (l/min) = 0.0308806 × Height (cm) − 2.877.

Boys: V′O_2_peak (l/min) = 0.044955 × Height (cm) − 4.64.

### 2.4. Statistical Methods

We performed a survival analysis through Cox Regression. Pulmonary exacerbation was considered an event, and survival time was measured from the first visit of the patient. All CPET indices were used as the relevant percentages of the predicted values.

Various combinations of CPET and other variables such as sputum culture, age, and spirometry measurements were tested via multivariate cox models.

All analyses were performed with statistical packages SPSS (IBM SPSS v22) and R (https://www.r-project.org/, date accessed on 12 June 2021) v3.5.2.

CPET variables’ relationship with exacerbations was visualized with Cox proportional hazard plot for the three groups of patients with VO_2_peak≤ 60% predicted; 60% < VO_2_peak% predicted ≤ 80%; and VO_2_peak% predicted >80%.

## 3. Results

Data from a 10-year follow up with CPET and spirometry of CF patients were used to perform our analyses. Baseline characteristics of patients included are shown on Table 1. Pulmonary exacerbations were recorded according to the above definition [12], and Cox proportional hazard models were applied.

### Cox Proportional Hazards Models

In the univariate analysis sputum culture, FEV_1_% predicted, FVC% predicted, VE/VO_2_, VE/VCO_2,_ and PetCO_2_ were found to be significant predictors of pulmonary exacerbations (Table 2). When adjusting the CPET models for age, sex, BMI, and sputum culture, the following parameters were found to be significant predictors of pulmonary exacerbations: VO_2_peak (Hazard ratio exp(B) (HR × B), 0.988 (0.978, 0.998) *p* = 0.019), VEVO_2_ (hazard ratio (HR × B), 1.033 (1.002, 1.065) *p* = 0.038), PetCO_2_ (HR × B 0.954 (0.917, 0.992) *p* = 0.017) and VO_2_max (Hazard ratio exp(B) (HR × B), 0.988 (0.979, 0.997) *p* = 0.007). (Table 3). After adjusting for FEV_1,_ VO_2_peak was also found to be significate predictor of upcoming exacerbations (HR × B), 0.988 (0.976, 0.999), *p* = 0.042 (Table 4); for each unit percent increase in VO_2_Peak, the relative risk of exacerbation is reduced by 1.2%.

**Figure 1:**
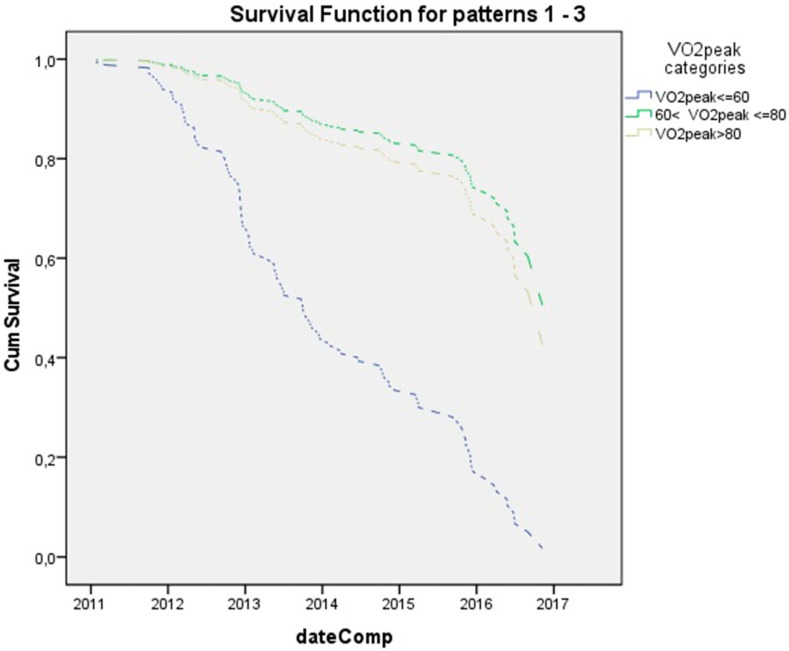
Kaplan-Meier survival curve until first PEx for the three VO_2_peak groups’ graph of cumulative survival, for each category of VO_2_peak, against time (years since entrance at the mean values of the covariates). Number of patients in each category. VO_2_peak ≤ 60%predicted *n* = 7, 60% < VO_2_peak %predicted ≤ 80% *n* = 23, and VO_2_peak > 80%predicted *n* = 48.

Furthermore, patients were divided into 3 categories according to their exercise capacity (patients with VO_2_peak < 60%, patients with 60% < VO_2_peak < 80%, and patients with VO_2_peak > 80% predicted). Patients in the two higher VO_2_peak groups had 4.2- and 4.5-times lower relative risk of having a pulmonary exacerbation than those at the low VO_2_peak group (*p* = 0.007 and *p* = 0.005, respectively) (Figure 1).

Similarly, the patients were divided into 3 groups according to their VE/VO_2_ and PETCO_2_, to assess them as a predictor of exacerbations.

**Figure 2:**
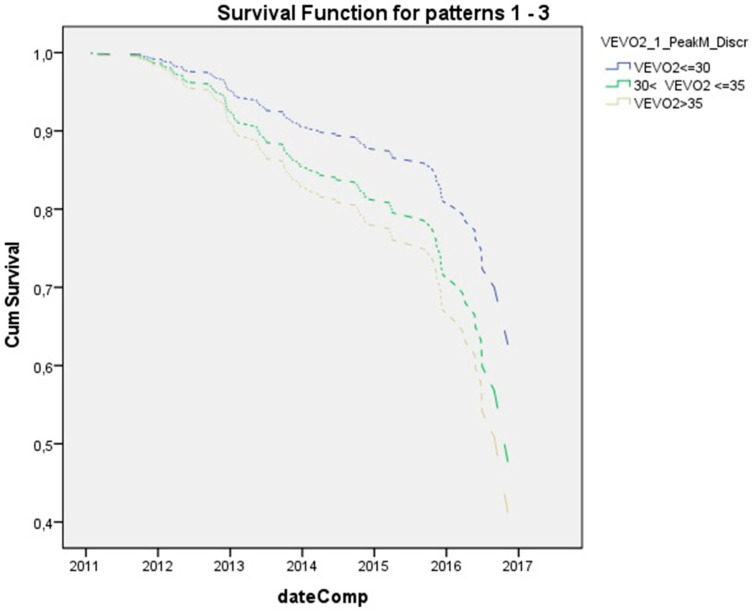
Hazard for exacerbation for three VEVO_2_ groups (VE/VO_2_ ≤ 30, 30 < VE/VO_2_ ≤ 35, and VE/VO_2_ > 35). Number of patients in each category: VE/VO_2_ ≤ 30 *n* = 8, 30 < VE/VO_2_ ≤ 35 *n* = 33, and VE/VO_2_ > 35 *n* = 37.

**Figure 3:**
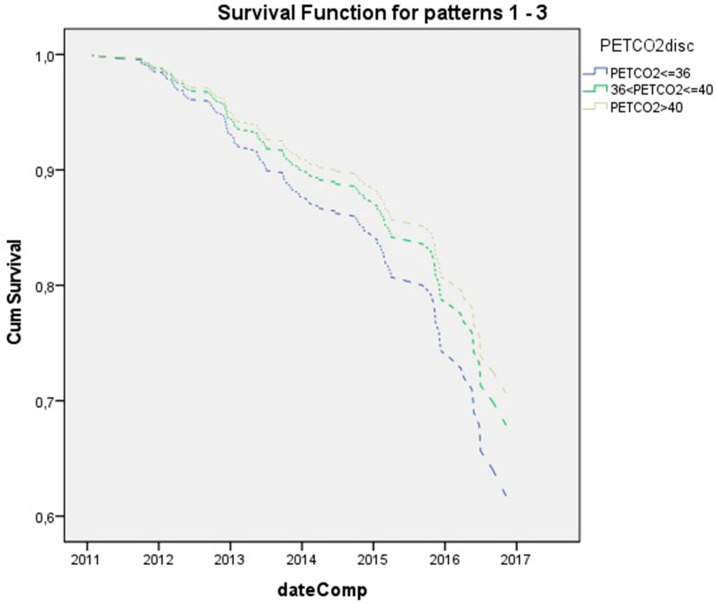
Kaplan-Meier survival curve until first PEx for three PETCO_2_ groups (PETCO_2_ ≤ 36, 36 < PETCO_2_ ≤ 40, and PETCO_2_ > 40).PETCO_2_ ≤ 36 *n* = 20, 36 < PETCO_2_ ≤ 40 *n* = 26, and PETCO_2_ > 40 *n* = 20.

Patients with VE/VO_2_ (≤30) and (>30 VE/VO_2_ ≤ 35) presented 0.8 and 0.5 times lower relative risk than the high (>35) group, though these differences were not statistically significant (*p* = 0.3 and *p* = 0.103, respectively) (Figure 2). Moreover, patients with PETCO_2_ (≤36) and (>36 VE/VO_2_ ≤ 40) displayed 1.4 and 1.1 times higher relative risk than the high (>40) category, though again these differences were not statistically significant (*p* = 0.13 and *p* = 0.6 correspondingly), (Figure 3).

## 4. Discussion

The main finding of this study is that Cardio-Pulmonary Exercise Testing can predict pulmonary exacerbations in patients with CF. To our knowledge, this is the first time this has been reported in the literature.

In recent years, CPET has gained increased interest among researchers in the field of respiratory disorders, especially cystic fibrosis. Since Nixon et al. first reported that CPET could predict mortality, many researchers have focused on confirming this finding [2]. Hebestreit et al. conducted multicenter research to examine whether CPET could predict mortality or lung transplant in a 10-year period [16]. The researchers found not only VO_2_peak but also ventilatory inefficiency indexes VE/VO_2_ and VE/VCO_2_ to be strong predictors of upcoming mortality. Another study employing univariate models recognized VE/VO_2_peak [3] as an indicator of mortality in adults.

Extending this concept, one could deduct that as these indices can predict mortality they could probably be also used as indicators for the events that lead to death in these patients’ pulmonary exacerbations. VE/VO_2_peak and VE/VCO_2_peak have been found to reflect structural lung damages, as noted on high-resolution computed tomography (HRCT) [17], and to be indicators of ventilatory inefficiency [18]. Chronic lung inflammation and remodeling are part of the mechanisms that lead to increased mortality in CF [19]. As airway inflammation and remodeling have progressed, ventilation inhomogeneity and ventilation inefficiency have become established [20]. This parallel progression can imply that both inflammation and ventilatory inefficiency might be related. Hebestreit el al. recognized VE/VO_2_ and VE/VCO_2_—the main ventilatory efficiency indexes—as prognostic factors for mortality [16].

In our study, and after multivariate analysis and adjustment, we recognized VE/VO_2_ along with PetCO_2_ and VO_2_peak as predictors of pulmonary exacerbation (PEx) in patients with CF. Moorcroft et al. recognized VE/VO_2_peak as a strong predictor of mortality in adult patients with CF [3], and it was also found to be a strong mortality predictor in children [5]. This prognostic importance of the ventilation efficiency index is strengthened in our study as it is found to be indicative not only of death but of the preceding pulmonary exacerbations that increase the disease burden in CF and eventually lead to death. Along with VE/VO_2_, end-tidal CO_2_ exhalation and VO_2_peak were found to be significant predictors of pulmonary exacerbations in CF, highlighting the role of CPET in monitoring disease severity and assessing upcoming disease exacerbations.

When looking at patients regarding their aerobic capacity, patients with VO_2_peak < 60% predicted showed about 80% more risk in developing a PEx during the following months in comparison to patients with VO_2_peak > 60% predicted. This is noted for the first time in literature and is of great clinical importance. Aerobic capacity is measured by a maximal cardiopulmonary exercise test with VO_2_peak—the amount of oxygen a person’s lungs absorbs during maximal exercise. Low aerobic capacity can be due to severe disease or deconditioning [21]. In CF, VO_2_peak < 60% predicted has been associated with poor survival [2,6]. The results of our study suggest that not only is low aerobic capacity is associated with worse prognosis but it can lead to more frequent exacerbations as well. In other words, the less fit a patient seems to be, the more prone to exacerbations he is. Hence, regular estimation of a patient’s exercise capacity helps identify those in danger of exacerbations. Even though the European Cystic Fibrosis Society recommends CPET as the gold standard method of assessing aerobic capacity, neither do all CF centers have CPET equipment nor can all patients undergo an exercise test periodically [22]. In this context, the findings of this study should not be considered as a mandate on performing CPETs but more as an encouragement to identify patients early on suspected of presenting low aerobic capacity. Even if CPET equipment is not available, other methods too [23,24,25,26] can allow for a rough assessment of a subject’s fitness levels. By recognizing patients with poor fitness, exercise interventions could be initiated. Preliminary data have shown that exercise training in CF patients can improve exercise capacity [16,27], whereas implementation of physical conditioning programs [16,28,29] along with escalation of medical treatment [30] can lead to avoidance of exacerbations. However, even though data on how physical training interventions can improve exercise capacity in CF have been published, there is a lack of evidence-based trials that substantiate these early findings [31], and future research should probably focus more to the merits of exercise in reducing exacerbation risk.

We observed that patients with 60 < VO_2_peak < 80% predicted at some points presented less possibility than those with VO_2_peak > 80% predicted, a finding that comes as a surprise and to our knowledge could not be attributed to anything. However, it must be noted that there is no statistically significant difference between the two as is to the first category of VO_2_peak < 60%.

## 5. Conclusions

Data from this 10-year single-center study show that VE/VO_2_, PetCO_2_, and VO_2_peak are significant predictors of pulmonary exacerbations in CF. Patients with low aerobic capacity present 4.5-times higher risk of developing a pulmonary exacerbation. CPET not only can provide data on mortality but also on upcoming exacerbations. The finding that lower exercise capacity is associated with an increased likelihood of exacerbation can prove of great help in everyday CF clinical care. In this context, motivating CF patients to maintain high fitness levels can lead to fewer pulmonary exacerbations and better quality of life.

## Figures and Tables

**Table 1:** Baseline characteristics of patients included.

Variable	Mean	SD
Age, years	14, 9	4, 7
Βody Mass Index (BMI), kg/m^2^	19, 6	3, 3
Height cm	153, 7	14, 4
Weight kg	47, 2	13, 4

SD: Standard deviation.

**Table 2:** Univariate models of Cardiopulmonary Exercise Testing (CPET) Predictors.

Variable	Relative Hazard (95% CI)	*p*-Value
Age, years	0.878, (0.851, 0.906)	**<0.001**
ΒΜΙ, kg/m^2^	0.872 (0.842, 0.903)	**<0.001**
Gender	1.171 (0.88, 1.56)	0.279
Sputum culture	0.314 (0.227, 0.433)	**<0.001**
FEV_1_% (%predicted)	0.99 (0.984, 0.996)	**0.002**
FVC% (%predicted)	0.98 (0.972, 0.988)	**<0.001**
VO_2_peak % (%predicted)	1.003 (0.994, 1.012)	0.536
VO_2_max % (%predicted)	0.993 (0.984, 1.02)	0.147
VE/VO_2_peak (Peak Ex)	1.037 (1.004, 1.071)	**0.029**
VE/VCO_2_peak (Peak Ex)	1.027 (1.001, 1.055)	**0.045**
PetCO_2_	0.96 (0.923. 0.999)	**0.045**

**Table 3:** Multivariate models of Pulmonary Exacerbations (PEx) prediction.

	CPET Variable				
CPET variables	**VO_2_peak**	**VE/VO_2_ Peak**	**VE/VCO_2_ Peak**	**PetCO_2_**	**VO_2_max**
	0.988 (0.978, 0.998) ***p* = 0.019**	1.033 (1.002, 1.065) ***p* = 0.038**	1.015 (0.992, 1.039) *p* = 0.213	0.954 (0.917, 0.992) ***p* = 0.017**	0.988 (0.979, 0.997) ***p* = 0.007**
Age	0.918 (0.886, 0.951) ***p* < 0.001**	0.929 (0.897, 0.961) ***p* < 0.001**	0.931 (0.900, 0.964) ***p* < 0.001**	0.929 (0.897, 0.917) ***p* < 0.001**	0.926 (0.895, 0.958) ***p* < 0.001**
Culture	0.451 (0.303, 0.672) ***p* < 0.001**	0.492 (0.328, 7.39) ***p* = 0.001**	0.479 (0.320, 0.717) ***p* < 0.001**	0.468 (0.313, 0.701) ***p* < 0.001**	0.461 (0.310, 0.686) ***p* < 0.001**

Five multivariate models for risk prediction are presented. Each model corresponds to a CPET variable and FEV is a covariate. In each model, we adjust for gender, age, BMI, and culture. The multivariate analyses adjusted for age, gender, BMI, and sputum culture demonstrated VO_2_peak along with VE/VO_2_ to be predictors of PEx. Significant correlations are highlighted in bold.

**Table 4:** Multivariate models of Pulmonary Exacerbations (PEx) prediction, adjusted for FEV_1_.

	CPET Variable				
CPET variables	**VO_2_peak**	**VE/VO_2_ peak**	**VE/VCO_2_ peak**	**PetCO_2_**	**VO_2_max**
	0.988 (0.976, 0.999) ***p* = 0.042**	1.002 (0.967, 1.038) *p* = 0.919	0.980 (0.949, 1.011) *p* = 0.2	0.964 (0.927, 1.003) *p* = 0.067	0.994 (0.985, 1.004) *p* = 0.244
Age	0.846 (0.810, 0.885) ***p* < 0.001**	0.860 (0.824, 0. 897) ***p* < 0.001**	0.853 (0.816, 0.891) ***p* < 0.001**	0.862 (0.826, 0.899) ***p* < 0.001**	0.861 (0.826, 0.962) ***p* < 0.001**
Culture	0.597 (0.390, 0.912) ***p* = 0.017**	0.492 (0.328, 7.39) ***p* = 0.001**	0.648 (0.423, 0.991) ***p* = 0.045**	0.633 (0.413, 0.970) ***p* = 0.036**	0.632 (0.415, 0.962) ***p* = 0.032**
FEV1	0.980 (0.977, 0.990) ***p* < 0.001**	0.492 (0.328, 7.39) ***p* = 0.001**	0.977 (0.967, 0.987) ***p* < 0.001**	0.980 (0.970, 0.989) ***p* < 0.001**	0.981 (0.972, 0.990) ***p* < 0.001**

After adjusting for FEV1 only VO_2_peak is statistically significant. We can state that for each unit percent that VO_2_peak is elevated the relative risk of exacerbation is reduced by 1.2%. Significant correlations are highlighted in bold.

## References

[1] Radtke T., Crook S., Kaltsakas G., Louvaris Z., Berton D., Urquhart D.S., Kampouras A., Rabinovich R.A., Verges S., Kontopidis D. (2019). ERS statement on standardisation of cardiopulmonary exercise testing in chronic lung diseases. Eur. Respir. Rev..

[2] Nixon P.A., Orenstein D.M., Kelsey S.F., Doershuk C.F. (1992). The prognostic value of exercise testing in patients with cystic fibrosis. N. Engl. J. Med..

[3] Moorcroft A.J., Dodd M.E., Webb A.K. (1997). Exercise testing and prognosis in adult cystic fibrosis. Thorax.

[4] Pianosi P., Leblanc J., Almudevar A. (2005). Peak oxygen uptake and mortality in children with cystic fibrosis. Thorax.

[5] Hulzebos E.H., Bomhof-Roordink H., van de Weert-van Leeuwen P.B., Twisk J.W., Arets H.G., van der Ent C.K., Takken T. (2014). Prediction of mortality in adolescents with cystic fibrosis. Med. Sci. Sports Exerc..

[6] Hebestreit H., Hulzebos E.H., Schneiderman J.E., Karila C., Boas S.R., Kriemler S., Dwyer T., Sahlberg M., Urquhart D.S., Lands L.C. (2019). Cardiopulmonary Exercise Testing Provides Additional Prognostic Information in Cystic Fibrosis. Am. J. Respir. Crit. Care Med..

[7] Urquhart D.S. (2011). Exercise testing in cystic fibrosis: Why (and how)?. J. R. Soc. Med..

[8] Villaverde-Hueso A., Sanchez-Diaz G., Molina-Cabrero F.J., Gallego E., Posada de la Paz M., Alonso-Ferreira V. (2019). Mortality Due to Cystic Fibrosis over a 36-Year Period in Spain: Time Trends and Geographic Variations. Int. J. Environ. Res. Public Health.

[9] Sanders D.B., Ostrenga J.S., Rosenfeld M., Fink A.K., Schechter M.S., Sawicki G.S., Flume P.A., Morgan W.J. (2020). Predictors of pulmonary exacerbation treatment in cystic fibrosis. J. Cyst. Fibros..

[10] Sanders D.B., Solomon G.M., Beckett V.V., West N.E., Daines C.L., Heltshe S.L., Dasenbrook E.C.D., van Decanter D.R., Solomon G.M., Goss C.H. (2017). Standardized Treatment of Pulmonary Exacerbations (STOP) study: Observations at the initiation of intravenous antibiotics for cystic fibrosis pulmonary exacerbations. J. Cyst. Fibros..

[11] Vermeulen F., Proesmans M., Boon M., Havermans T., De Boeck K. (2014). Lung clearance index predicts pulmonary exacerbations in young patients with cystic fibrosis. Thorax.

[12] Bilton D., Canny G., Conway S., Dumcius S., Hjelte L., Proesmans M., Tümmler B., Vavrova V., De Boeck K. (2011). Pulmonary exacerbation: Towards a definition for use in clinical trials. Report from the EuroCareCF Working Group on outcome parameters in clinical trials. J. Cyst. Fibros..

[13] Beydon N., Davis S.D., Lombardi E., Allen J.L., Arets H.G.M., Aurora P., Bisgaard H., Davis G.M., Ducharme F.M., Eigen H. (2007). An official American Thoracic Society/European Respiratory Society statement: Pulmonary function testing in preschool children. Am. J. Respir. Crit. Care Med..

[14] Godfrey S., Davies C.T., Wozniak E., Barnes C.A. (1971). Cardio-respiratory response to exercise in normal children. Clin. Sci..

[15] Orenstein D. (1993). Assessment of Exercise Pulmonary Function.

[16] Hebestreit H., Kieser S., Junge S., Ballmann M., Hebestreit A., Schindler C., Shcenk T., Posselt H.-G., Kriemler S. (2010). Long-term effects of a partially supervised conditioning programme in cystic fibrosis. Eur. Respir. J..

[17] Hatziagorou E., Kampouras A., Avramidou V., Georgopoulou V., Kirvasilis F., Kontouli K., Hebestreit H., Tsanakas J. (2016). Exercise responses are related to structural lung damage in CF pulmonary disease. Pediatr. Pulmonol..

[18] Kampouras A., Hatziagorou E., Avramidou V., Georgopoulou V., Kirvassilis F., Hebestreit H., Tsanakas J. (2019). Ventilation efficiency to exercise in patients with cystic fibrosis. Pediatr. Pulmonol..

[19] De Bentzmann S., Roger P., Puchelle E. (1996). Pseudomonas aeruginosa adherence to remodelling respiratory epithelium. Eur. Respir. J..

[20] Di Paolo M., Teopompi E., Savi D., Crisafulli E., Longo C., Tzani P., Longo F., Ielpo A., Pisi G., Cimino G. (2019). Reduced exercise ventilatory efficiency in adults with cystic fibrosis and normal to moderately impaired lung function. J. Appl. Physiol..

[21] Argo C.K., Stine J.G., Henry Z.H., Lackner C., Patrie J.T., Weltman A.L., Caldwell S.H. (2018). Physical deconditioning is the common denominator in both obese and overweight subjects with nonalcoholic steatohepatitis. Aliment. Pharmacol. Ther..

[22] Barker M., Hebestreit A., Gruber W., Hebestreit H. (2004). Exercise testing and training in German CF centers. Pediatr. Pulmonol..

[23] Werkman M.S., Hulzebos E.H., Helders P.J., Arets B.G., Takken T. (2014). Estimating peak oxygen uptake in adolescents with cystic fibrosis. Arch. Dis. Child..

[24] Radtke T., Puhan M.A., Hebestreit H., Kriemler S. (2016). The 1-min sit-to-stand test—A simple functional capacity test in cystic fibrosis?. J. Cyst. Fibros..

[25] Lang R.L., Stockton K., Wilson C., Russell T.G., Johnston L.M. (2020). Exercise testing for children with cystic fibrosis: A systematic review. Pediatr. Pulmonol..

[26] Andrade Lima C., Dornelas de Andrade A., Campos S.L., Brandão D.C., Mourato I.P., Britto M.C.A. (2018). Six-minute walk test as a determinant of the functional capacity of children and adolescents with cystic fibrosis: A systematic review. Respir. Med..

[27] Selvadurai H.C., Blimkie C.J., Meyers N., Mellis C.M., Cooper P.J., Van Asperen P.P. (2002). Randomized controlled study of in-hospital exercise training programs in children with cystic fibrosis. Pediatr. Pulmonol..

[28] Kriemler S., Kieser S., Junge S., Ballmann M., Hebestreit A., Schindler C., Stüssi C., Hebestreit H. (2013). Effect of supervised training on FEV1 in cystic fibrosis: A randomised controlled trial. J. Cyst. Fibros..

[29] Kriemler S., Radtke T., Christen G., Kerstan-Huber M., Hebestreit H. (2016). Short-Term Effect of Different Physical Exercises and Physiotherapy Combinations on Sputum Expectoration, Oxygen Saturation, and Lung Function in Young Patients with Cystic Fibrosis. Lung.

[30] Bhatt J.M. (2013). Treatment of pulmonary exacerbations in cystic fibrosis. Eur. Respir. Rev..

[31] Hebestreit H., Lands L.C., Alarie N., Schaeff J., Karila C., Orenstein D.M., Urquhart D.S., Hulzebos E.H.J., Stein L., the ACTIVATE-CF Study Working Group (2018). Effects of a partially supervised conditioning programme in cystic fibrosis: An international multi-centre randomised controlled trial (ACTIVATE-CF): Study protocol. BMC Pulm Med..

